# Kiwifruit-*Agaricus blazei* intercropping effectively improved yield productivity, nutrient uptake, and rhizospheric bacterial community

**DOI:** 10.1038/s41598-024-66030-z

**Published:** 2024-07-17

**Authors:** Chuan Shen, Xia Li, Jianfeng Qin

**Affiliations:** 1 Shaannan Eco-Economy Research Center, Ankang University, Ankang, 725000 China; 2 Department of Electronic and Information Engineering, Ankang University, Ankang, 725000 China; 3 Ankang Academy of Agricultural Sciences, Ankang, 725000 China

**Keywords:** Intercropping, Kiwifruit, *Agaricus blazei*, Soil properties, Bacterial community, Microbiology, Molecular biology, Plant sciences

## Abstract

Intercropping systems have garnered attention as a sustainable agricultural approach for efficient land use, increased ecological diversity in farmland, and enhanced crop yields. This study examined the effect of intercropping on the kiwifruit rhizosphere to gain a deeper understanding of the relationships between cover plants and kiwifruit in this sustainable agricultural system. Soil physicochemical properties and bacterial communities were analyzed using the Kiwifruit-*Agaricus blazei* intercropping System. Moreover, a combined analysis of 16S rRNA gene sequencing and metabolomic sequencing was used to identify differential microbes and metabolites in the rhizosphere. Intercropping led to an increase in soil physicochemical and enzyme activity, as well as re-shaping the bacterial community and increasing microbial diversity. Proteobacteria, Bacteroidota, Myxococcota, and Patescibacteria were the most abundant and diverse phyla in the intercropping system. Expression analysis further revealed that the bacterial genera BIrii41, Acidibacter, and Altererythrobacter were significantly upregulated in the intercropping system. Moreover, 358 differential metabolites (DMs) were identified between the monocropping and intercropping cultivation patterns, with fatty acyls, carboxylic acids and derivatives, and organooxygen compounds being significantly upregulated in the intercropping system. The KEGG metabolic pathways further revealed considerable enrichment of DMs in ABC transporters, histidine metabolism, and pyrimidine metabolism. This study identified a significant correlation between 95 bacterial genera and 79 soil metabolites, and an interactive network was constructed to explore the relationships between these differential microbes and metabolites in the rhizosphere. This study demonstrated that Kiwifruit-*Agaricus blazei* intercropping can be an effective, labor-saving, economic, and sustainable practice for reshaping bacterial communities and promoting the accumulation and metabolism of beneficial microorganisms in the rhizosphere.

## Introduction

The involvement of plant-soil feedback responses in mechanisms related to various ecological phenomena has gained increasing attention. These responses encompass both positive and negative interactions between plants and soil organisms^1^. Rhizospheres are microdomains surrounding plant roots that contain a diverse microbial community and are particularly active in plant–microbe interactions, significantly affecting plant growth and health maintenance^2^. The relationship between plant growth and vitality and the microbial community in the rhizosphere is multifaceted and influenced by both soil factors and plant characteristics, which together contribute to the plant's overall vitality and growth^3^.

However, the advent of intensive agricultural practices, characterized by the excessive use of pesticides and chemical fertilizers, has precipitated significant environmental concerns, including water and air pollution. Moreover, the practice of monoculture cropping, especially with horticultural crops, has been implicated in the degradation of soil quality, a phenomenon referred to as soil sickness^4^. This degradation not only diminishes crop yield and quality but also exacerbates the vulnerability of crops to diseases and pests^5,6^. In this context, exploring sustainable agricultural practices such as intercropping and the use of cover crops becomes imperative. These practices not only mitigate the negative impacts of monoculture but also enhance soil fertility and biodiversity, thereby promoting a more sustainable and productive agricultural system.

Several strategies for growing crops simultaneously in combination, such as intercropping, are considered alternatives to increasing the quality of monoculture soils^7^. Research has shown that intercropping can enhance microbial diversity and abundance, leading to improved soil structure, increased availability of nutrients, and enhanced disease suppression^8^. The root exudates from different plants in an intercropping system can attract distinct microbial populations, fostering a more complex and resilient microbial ecosystem^9^. This, in turn, can enhance nutrient uptake by plants and contribute to the suppression of soil-borne pathogens through the promotion of beneficial microbes^10^. For example, intercropping has a favorable influence on soil characteristics compared to maize monoculture and improves the richness and variety of soil bacteria at the taxonomic genus level^11^. Furthermore, intercropping has been observed to influence the balance between different microbial taxa, which can have profound effects on the biochemical processes within the soil. For example, the increased nitrogen fixation by leguminous plants in certain intercropping systems can enrich soil nitrogen content, benefiting both the crops and the microbial communities that rely on nitrogen for their metabolic processes^12^.

Kiwifruit (*Actinidia chinensis*) is a perennial deciduous vine of the genus *Actinidia* (Lindl.), is a well-known fruit with a high content of vitamin C, flavonoids, carotenoids, and dietary fiber, and is considered the "king of fruits" with a high commercial value^13^. However, it requires a stringent growth environment because its growth and development processes are vulnerable to various biotic and abiotic stresses, such as diseases, pests, drought, low nutrition, and weeds. Notably, consecutive years of agricultural practices and the prevailing management strategy inherent in the traditional clear-cut system have precipitated a range of ecological and environmental challenges associated with kiwifruit cultivation. These challenges include, but are not limited to, soil degradation, the depletion of nutrients, and environmental contamination^14,15^. The ability to enhance the inherent resistance of kiwifruit against external stressors is of critical importance. Moreover, the diversity of beneficial rhizosphere microorganisms can greatly contribute to the improvement of kiwifruit's resistance.

Recent studies have highlighted the potential of intercropping systems, involving kiwifruit and various other crops, such as *Amorphophallus konjac*, *Trifolium repens, Lupin spp., Vulpia myuros, Astragalus spp.* (Milk vetch)*, Lolium perenne* L., *Vicia sativa* L., *Reineckea carnea*, etc.^16–18^. These systems have been found to significantly enhance soil quality, augment microbial diversity, and improve nutrient availability in the rhizosphere, consequently boosting both the yield and quality of kiwifruit. In particular, intercropping with *Vicia sativa* L. and *Reineckea carnea* has been shown to significantly improve the moisture content, microbial community, enzyme activity, and nutrient levels in the rhizosphere soils of kiwifruit plants, thereby enhancing the yield and quality of kiwifruit. These findings suggest that intercropping can improve soil quality and microbial activity by increasing moisture content, fertility, and rhizosphere structure as well as suppressing weeds to foster plant growth and development with increased profitability^19–21^. Furthermore, intercropping emerges as a strategic approach to manage soil erosion and optimize resource utilization, thereby increasing land and labor productivity. This approach facilitates efficient, high-quality production and ecological preservation in kiwifruit orchards. However, it is imperative to explore a broader range of crops such as mushroom, suitable for intercropping with kiwifruit, particularly those with short growth cycles that can provide nutrients to the kiwifruit and assist in maintaining soil moisture levels.

The cultivation of cover crops within orchards represents a contemporary strategy for preserving soil integrity, with empirical evidence underscoring their critical role in mitigating soil and water erosion, augmenting soil nutrient content, and enhancing microbial activity^22–24^. Among these sustainable practices, *Agaricus blazei* Murrill (*A. blazei*), is a widely cultivated medicinal mushroom with well-documented therapeutic properties and a long history of traditional use^25^. This species is rich in bioactive compounds known for their potent antimicrobial properties. In various Eastern nations, *A. blazei* is esteemed not only for its gastronomic attributes but also for its prospective health benefits, particularly in cancer prevention and therapy^26,27^. Remarkable progress has been made in the cultivation techniques of *A. blazei*, and the species can be intercropped in orchards. Previous research has demonstrated that *A. blazei* can be used as a biofertilizer, enhancing moisture conservation, crop yields, and the quality of intercropped plants^28^*.* Consequently, we posited that the intercropping of A. blazei could influence the development of kiwifruit by facilitating the root system's absorption and translocation of substances beneficial to plant growth. Moreover, the introduction of cover crops and biofertilizers, such as *A. blazei*, into kiwifruit orchards introduces an innovative method for sustaining soil vitality and advancing sustainable agricultural modalities. Nonetheless, the specific impacts of A. blazei intercropping on the structural composition of soil microflora and nutrient concentrations in orchard soils remain unexplored.

In the present study, we employed 16S rRNA gene sequencing and soil microbial LC–MS untargeted metabolic profiling to investigate the soil bacterial community structure and diversity in kiwifruit/*A. blazei* intercropping system. The results demonstrated that the physicochemical characteristics and microbial diversity were enhanced in the kiwifruit/*A. blazei* intercropping system compared to the monocropping system. These findings provide novel insights into the development of effective and economically viable kiwifruit cultivation practices.

## Materials and methods

### Plant materials and experimental design

**Figure 1:**
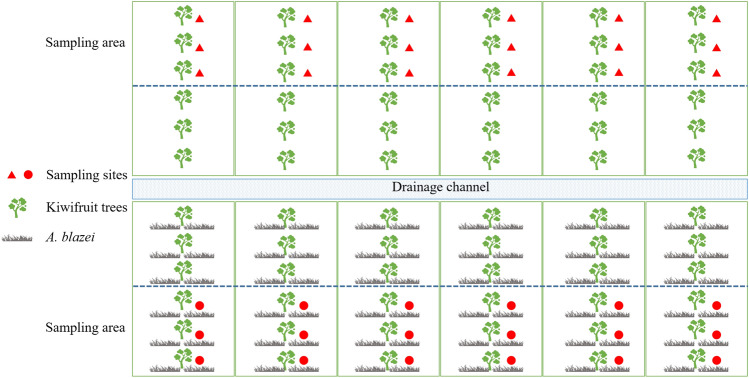
Conceptual representation of the experimental design adopted for intercropping in this study. Area 1 represents control in the upper part of the planting diagram and area 2 represent treat in the lower part of the planting diagram.

The field experiment in this study was conducted at a seedling trial site in Ankang, Shaanxi Province, China using the kiwifruit cultivar Jinyan in its sixth year of planting. The sample site was a kiwifruit orchard that had been planted continuously for six years and the intercropping with *A. blazei* was the first year. Accordingly, the field was divided into two equal sections for the treatment and control experiments. Experimental layout of kiwifruit/*A. blazei* intercropping system is shown in Fig. 1.

In May, soil samples were collected from the roots of kiwifruit trees at six representative sampling sites, with three biological replicates per sampling site. The samples from each replicate site were mixed together to form an experimental sample. Samples from sampling areas 1 and 2 were designated as control 1 and treat 1, respectively (Fig. 1). Subsequently, *A. blazei* was planted around the roots of kiwifruit trees, particularly in areas where soil samples were previously collected. The experimental field was kept moist until fruit ripening, and soil samples were collected in October. Samples from sampling areas 1 and 2 were designated as control 2 and treat 2, respectively (Fig. 1). The collected soil samples were analyzed for changes in soil microbiota, and kiwifruits were collected for physiological index determination.

The mixed experimental samples were filtered using a 2 mm sieve to remove impurities such as large stones. Each sample was then divided into three portions and immediately placed in liquid nitrogen before being transferred to a − 80 °C refrigerator. Two sample portions were used for soil microbiological and physicochemical property analyses, while the remaining portion was used as a backup.

### Determination of soil physicochemical properties and enzyme activity

The soil pH was determined using the potentiometric method (FE28-Meter). Soil organic carbon (SOC) and total nitrogen (TN) were estimated using the potassium dichromate oxidation capacity method (vario Macro cube) and sulfuric acid-accelerator decoction method (KjeltecTM 8400), respectively. The semi-micro Kjeldahl diffusion method was adopted to measure alkaline hydrolytic nitrogen (ALN). Similarly, available potassium (AK) and phosphorus (AP) in the soil were assessed using the ammonium acetate leaching-flame photometry method (PinA Aciie 900F) and soil rapid phosphorus content method, which involves sodium bicarbonate leaching and molybdenum antimony colorimetry (UV-2450), respectively. Microbial biomass carbon (MBC) and nitrogen (MBN) in the soil were determined using the chloroform fumigation-extraction method (TOC-VCPH; MBC and Auto Analyzer 3-AA3; MBN)^29^.

The soil enzyme activity was measured using a reagent kit from the Nanjing Jiancheng Institute of Biological Engineering. Different colorimetry methods were employed to estimate urease (URE; sodium phenol-sodium hypochlorite method), sucrase (INV; 3,5-dinitrosalicylic acid method), alkaline phosphatase (ALP; sodium benzene phosphate method) and nitrate reductase (NR; phenol-disulfonic acid method) activities in the soil. Catalase (CAT) activity was determined using UV spectrophotometry.

### Determination of kiwifruit quality

The weight of each kiwifruit was measured using a balance scale. The contents of soluble solids and total acid, along with the solid-to-acid ratio, were determined using a hand-held sugar and acid integrator (ATAGO, PAL-BX/ACID 8, Japan). Similarly, the soluble sugar and titratable acid contents were estimated using the respective anthrone colorimetric and NaOH neutralization methods to calculate the sugar-acid ratio. Furthermore, the vitamin C content of kiwifruit was analyzed using the 2,6-dichloroindophenol titration method, and the dry matter content was estimated using the oven-drying method.

### DNA extraction, PCR amplification and Illumina MiSeq sequencing

Microbial DNA was extracted from 0.5 g soil samples and purified using the E.Z.N.A. Soil DNA kit (Omega Bio-Tek, USA). DNA quality and concentration were confirmed using NanoDrop spectrophotometer and agarose gel electrophoresis. Extracted DNA was diluted to a concentration of 1 ng/L using sterile water and kept at − 20 °C until further processing. For bacterial amplicon sequencing and diversity analysis, V3-V4 variable regions of 16S rRNA genes were PCR-amplified using diluted DNA as a template with the universal primers 343F (5′-TACGGRAGGCAGCAG-3′) and 798R (5′-AGGGTATCTAATCCT-3′)^30^ using Takara Ex Taq (Takara, China) according to the manufacturer’s instructions. 16S amplicon sequencing and analysis were performed by OE Biotech Co., Ltd. (Shanghai, China). Subsequently, the TruSeq® DNA PCR-Free Sample Preparation Kit (Illumina, United States) was used to generate sequencing libraries following the manufacturer's recommendations. Finally, the libraries were sequenced on an Illumina NovaSeq6000 platform with two paired-end read cycles of 250 bases each.

Raw data were submitted to the QIIME2 (Quantitative Insights into Microbial Ecology version 2) system to filter, trim, and merge the demultiplexed sequences with DADA2, followed by the removal of detected chimeric sequences. First, the representative read of each amplicon sequence variant (ASV) was selected using the QIIME2 platform^31^. Next, a representative set of reads was annotated and blasted against the Silva database (https://ftp.arb-silva.de) using the default configuration of the q2-feature-classifier. Finally, analysis operations, such as alpha and beta diversity analysis and microbial multivariate statistical analysis, were carried out on the cloud platform (https://cloud.oebiotech.cn/task/). Based on the Bray–Curtis distance, a Principal Coordinates Analysis (PCoA) was utilized to visualize the change in community makeup. Using the "betadisper" and "adonis" functions, a permutational multivariate analysis of variance (PERMANOVA) was carried out to compare the differences between the cropping systems. Functional analysis of the bacterial community was performed using the PICRUSt2 (phylogenetic investigation of communities by reconstruction of unobserved states) algorithm^32^.

### Metabolome sample preparation and liquid chromatography–mass spectrometry (LC–MS) analysis

20 μL of internal standard and 1 mL of methanol–water (v/v; 1:1) mixture were added to 500 mg of the soil sample. In the consecutive step, 150 μL of supernatant was aspirated with a syringe, filtered using a 0.22 μm organic phase pinhole filter, transferred to LC injection vials, and stored at − 80 °C until LC–MS analysis was performed.

LC–MS sequencing and analysis were performed by OE Biotech Co., Ltd. (Shanghai, China). Quality control (QC) samples were prepared by mixing equal volumes of extracts from all samples. Metabolomic profiling of samples was carried out using an LC–MS system equipped with an ACQUITY UPLC I-Class system (Waters Corporation, USA) and a VION IMS QT mass spectrometer (Waters Corporation, USA). Metabolic profiling analyses were conducted in both ESI positive and negative ion modes. Mobile phases A and B were composed of water and acetonitrile/methanol (2:3, v/v), and each contained 0.1% formic acid. The column temperature was 45 ℃, and the flow rate remained at 0.35 mL/min. Each sample was maintained at 4 °C throughout the analysis, and the injection volume was 2 μL. Full scan mode (ranging from 50 to 1000 m/z) was used to obtain the metabolite data.

### Metabolomics analysis based on LC–MS data

The LC–MS raw data were initially transferred to the software Progenesis QI V2.3 software (Nonlinear, UK) for baseline filtering, peak identification, retention time correction, peak alignment, and normalization. The Human Metabolome Database (HMDB), Lipidmaps, and Metlin databases were used for the qualitative analysis of compound identification based on the exact mass-to-charge ratio (m/z), secondary fragments, and isotopic distribution. The positive and negative ion data were combined into a data matrix and subjected to multivariate analysis.

Next, unsupervised principal component analysis (PCA) was conducted to monitor the overall distribution of the processing samples and the stability of the entire analysis process. Metabolic differences between groups were monitored using supervised orthogonal partial least-squares discriminant analysis (OPLS-DA) and partial least-squares discriminant analysis (PLS-DA). Additionally, the variable importance of projection (VIP) values derived from the OPLS-DA model were used to rank the overall contribution of each variable to group discrimination. Furthermore, a two-tailed Student's t-test was used to determine statistically significant metabolite differences between groups. VIP scores > 1.0 and p-values < 0.05 were used to identify critically differential metabolites.

### Statistical analyses

The experimental data were analyzed using SPSS 23.0 (Chicago, USA), and statistically significant differences were evaluated by one-way analysis of variance (ANOVA) and Duncan’s multiple range test (*p* < 0.05). The graphs were produced using Origin software version 9.1 (Origin Lab, USA).

## Results

### Kiwifruit/*A. blazei* intercropping enhanced soil physicochemical properties and enzyme activity

No statistically significant differences were observed between groups S1 and S3, indicating a lack of heterogeneity among the sampling areas (Table 1). Consequently, subsequent differential comparisons will focus primarily on the analysis of groups S2 and S4. Intercropping significantly affected soil physicochemical properties, with an approximately twofold enhancement in ALN, TN, and SOC levels during kiwifruit intercropping compared with monoculture or control soils. Likewise, the amount of AP increased more than threefold, while the increase in AK levels was insignificant. In addition, MBC and MBN values indicated more than double the growth in the total number of soil microorganisms. Although other physical and chemical properties changed markedly, the change in pH was negligible.

**Table 1:** Comparison of soil physicochemical properties in crop monoculture (CK) and intercropping with *Agaricus blazei* (IA) in kiwifruit orchard.

Samples		SOC	TN	ALN	AK	AP	MBC	MBN	pH
		g kg^−1^	g kg^−1^	mg kg^−1^	mg kg^−1^	mg kg^−1^	mg kg^−1^	mg kg^−1^	
CK	Control 1	16.44 ± 2.76 b	1.43 ± 0.32 b	122.85 ± 4.58 a	615.68 ± 58.26 a	264.47 ± 4.54 a	352.57 ± 26.86 b	38.06 ± 7.16 b	7.60 ± 0.17 a
	Control 2	13.08 ± 2.92 b	1.27 ± 0.20 b	53.08 ± 5.75 b	200.18 ± 22.90 b	62.67 ± 14.08 b	258.79 ± 33.51 b	30.72 ± 3.76 b	7.64 ± 0.05 a
IA	Treat 1	12.27 ± 5.54 b	1.06 ± 0.62 b	57.05 ± 6.13 b	289.46 ± 297.62 b	122.60 ± 121.30 b	270.86 ± 160.99 b	21.39 ± 13.24 b	7.57 ± 0.03 a
	Treat 2	25.76 ± 1.35 a	2.5 ± 0.08 a	113.17 ± 2.75 a	226.61 ± 12.46 b	194.33 ± 26.25 a	808.39 ± 100.33 a	73.10 ± 4.05 a	7.60 ± 0.09 a

*TN* total nitrogen, *SOC* soil organic carbon, *ALN* alkaline hydrolytic nitrogen, *AP* available phosphorus, *AK* available potassium, *MBC* soil microbial biomass carbon, *MBN* soil microbial biomass nitrogen. Control 1: samples collected in May; Control 2: samples collected in October; Treat 1: samples collected in May; Treat 2: samples collected in October. Different lower-case letters a and b indicate significant differences (*p* < 0.05). Values represent the means of three replicates ± SD.

Intercropping systems depend considerably on soil enzymes. Therefore, the activities of the five major soil enzymes were determined to identify those essential for the system (Table 2). No significant difference in the activities of INV, SP, and CAT were observed between the intercropping and monocropping soils. However, a nearly twofold enhancement in URE and NR activities was obtained in the intercropping cultivation compared to that in the monoculture cultivation.

**Table 2:** Activities of five major enzymes in the rhizosphere soils of crop monoculture (CK) and intercropping with *Agaricus blazei* (IA) cultivation.

Samples		URE	INV	SP	CAT	NR
		ug g h	ml g h	ug g h	mg g d	ug g h
CK	Control 1	22.21 ± 7.09 a	47.10 ± 6.96 a	10.24 ± 0.58 b	7.63 ± 0.02 a	0.61 ± 0.10 b
	Control 2	10.03 ± 2.16 b	53.04 ± 10.11 a	22.91 ± 2.40 a	8.30 ± 0.26 a	0.55 ± 0.11 b
IA	Treat 1	13.08 ± 5.16 b	45.06 ± 28.13 a	6.66 ± 1.19 b	7.84 ± 0.03 a	0.54 ± 0.23 b
	Treat 2	16.34 ± 2.30 a	59.09 ± 3.32 a	23.74 ± 2.27 a	8.54 ± 0.03 a	0.98 ± 0.08 a

*URE* urease, *INV* sucrase, *SP* soil phosphatase, *CAT* catalase, *NR* nitrate reductase. Control 1: samples collected in May; Control 2: samples collected in October; Treat 1: samples collected in May; Treat 2: samples collected in October. Different lower-case letters a and b indicate significant differences (*p* < 0.05). Values represent the mean ± standard deviation of three replicates.

### Changes in the soil microbial community at the phylum and genus levels driven by kiwifruit/*A. blazei* intercropping

The 16S rRNA gene sequence fragments from the 24 soil samples were examined to determine alterations in the microbial community induced by the intercropping system. For each sample, the sequencing raw read data ranged from 78,022 to 81,598, clean tags data following quality control ranged from 25,062 to 52,239, and clean tags data after removing chimeras to obtain valid tags (the final data for analysis) ranged from 23,869 to 50,330. In addition, the number of ASVs ranged from 648 to 1757 (Table S1). Finally, a total of 10,599 ASVs and a core set containing 48 ASVs were obtained (Fig. S1).

**Figure 2:**
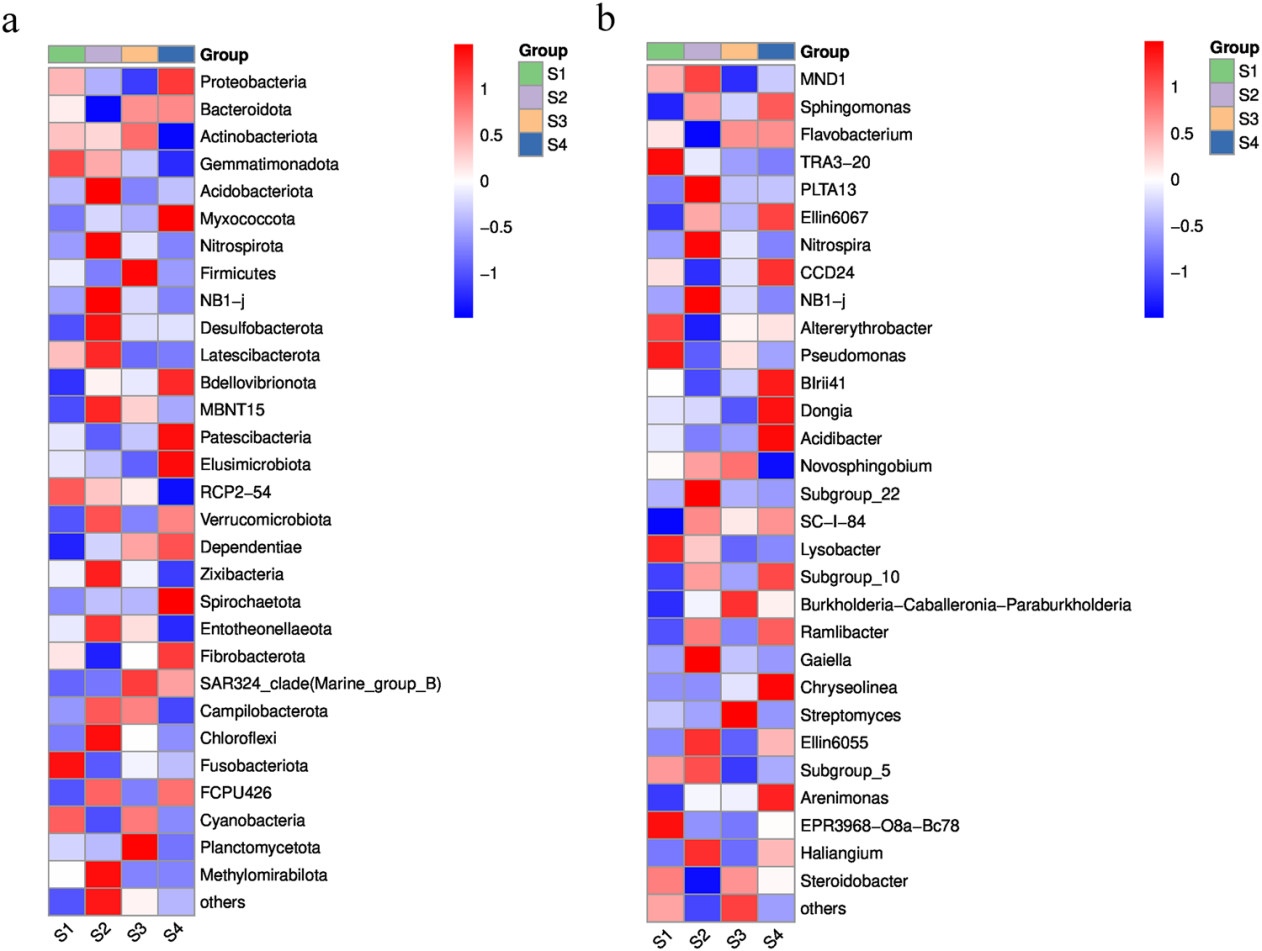
Bacterial-community composition under monocropping and intercropping cultivation patterns. (**a**) Heatmap analysis of the top 30 phyla in the rhizospheric soils. (**b**) Heatmap analysis of the top 30 genera in the rhizospheric soils. S1: Control 1; S2: Control 2; S3: Treat 1; S4: Treat 2.

The taxonomic distribution at the phylum level is shown in Fig. S2. Proteobacteria was observed to be the most abundant phylum, accounting for 47.65–56.73% of the total valid reads in all the samples. The second most abundant phylum, Bacteroidetes accounted for 10.63–23.42% of the total valid reads, with an average relative abundance of 16.46%. The other dominant phyla were Actinobacteriota (5.28–17.00%, average value of 8.49%), Gemmatimonadota (2.47–8.57%, average value of 5.63%), Acidobacteria (2.80–6.74%, average value of 4.70%), and Myxococcota (2.60–6.64%, average value of 3.73%). However, differences in the relative abundance of these phyla between the two cropping systems were observed. Notably, the proportions of Proteobacteria, Bacteroidetes, Myxococcota, Patescibacteria, Elusi_microbiota, and Bdellovibrionota in the rhizospheric soil samples of the intercropping systems were higher than those in the monocropping systems (Fig. 2a).

The most abundant bacterial genera in the rhizosphere soil of the intercropping system were *Flavobacterium*, *BIrii41*, and *Acidibacter* (Fig. 2b). In addition, the relative abundances of other dominant genera, such as *Chryseolinea*, *CCD24*, and *Altererythrobacter*, were significantly higher in the intercropping system than in the monoculture system. Considering these findings, it is evident that Kiwifruit/*A. blazei* intercropping system altered the microbial community, distinguishing it from the monocropping system.

### Kiwifruit/*A. blazei* intercropping enriched the structural and diversity of the soil microbial community

To analyze the degree of species diversity within the biological environment, alpha diversity was determined to describe the richness and diversity index of the bacterial community in the soil with multiple indices (Table 3). The Shannon index, which is positively linked with the diversity of the soil bacterial community, revealed the evenness of the soil bacterial community. The number of ACE and the Chao1 index differed remarkably between the two groups (monoculture and intercropping). However, no significant differences were observed in the Shannon index.

**Table 3:** Alpha diversity indices in crop monoculture (CK) and intercropping (IA) soils.

Treatments		ACE	Chao 1	Shannon
CK	Control 1	1215.66 ± 173.94 b	1218.97 ± 175.54 b	9.51 ± 0.35 a
	Control 2	1118.33 ± 130.64 b	1123.42 ± 121.00 b	9.40 ± 0.40 a
IA	Treat 1	1110.21 ± 136.63 b	1115.32 ± 137.02 b	9.32 ± 0.20 a
	Treat 2	1412.07 ± 113.49 a	1417.49 ± 112.83 a	9.64 ± 0.27 a

Variables: Control 1: samples collected in May; Control 2: samples collected in October; Treat 1: samples collected in May; Treat 2: samples collected in October. Different letters indicate significant differences (*p* < 0.05). Values represent the mean ± standard deviation of three replicates.

**Figure 3:**
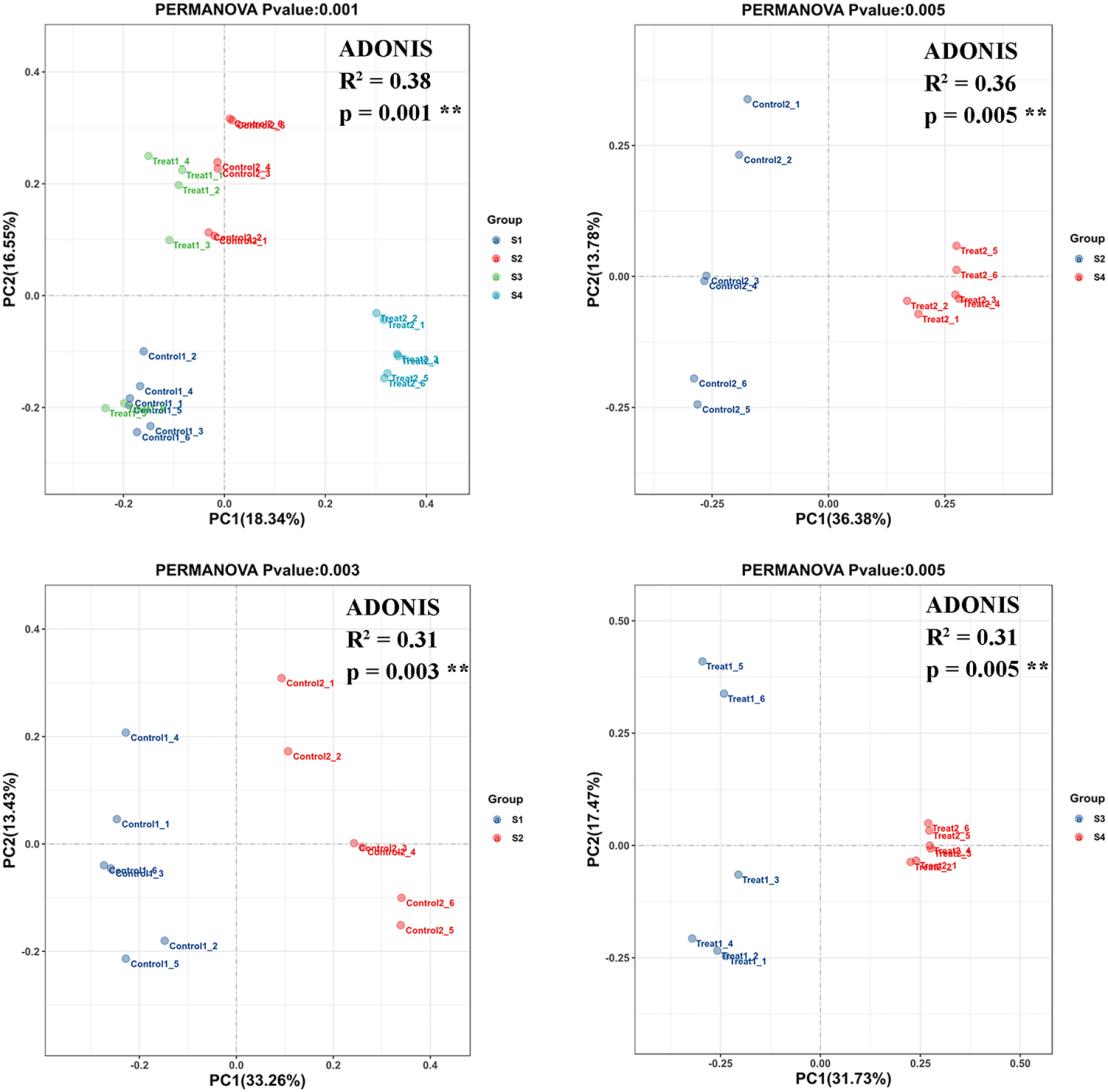
Principal coordinate analysis (PCoA) of bacterial communities based on Bray–Curtis similarity index and *P*-values (*P* < 0.01) determination based on ADONIS statistics in the rhizosphere soil of monoculture cropping and intercropping cultivation patterns. S1: control 1; S2: control 2; S3: treat 1; S4: treat 2.

The Bray–Curtis similarity index method was adopted to examine the difference in *p*-values of bacterial beta diversity at the ASV level. The *p*-values < 0.05 were considered statistically significant for subgroups. The R^2^ values indicate the degree of explanation of differences between samples in the subgroups. As shown in Fig. 3, principal coordinate analysis (PCoA) revealed the degree of separation of the soil samples. The results of permutational multivariate analysis of variance (PERMANOVA) indicated that intercropping with *A. blazei* in the kiwifruit orchard significantly altered the soil bacterial community (*p* < 0.01). ADONIS statistics showed an R^2^ value of 0.38 between different groups. Among these, an R^2^ value of 0.36 was obtained between intercropping and monocropping systems during the kiwifruit harvesting season.

### Differential analysis of soil microbial diversity

The Wilcoxon algorithm was used to calculate the statistical significance of the differences in soil microbes between the two planting patterns, with a default screening criterion of *p* < 0.05. The results indicated that among the top 10 microbial genera, the relative abundances of beneficial genera, such as *BIrii41*, *Acidibacter*, and *Altererythrobacter* were significantly different in the intercropping system than in the control monocropping system (*p* < 0.05) (Fig. S3).

**Figure 4:**
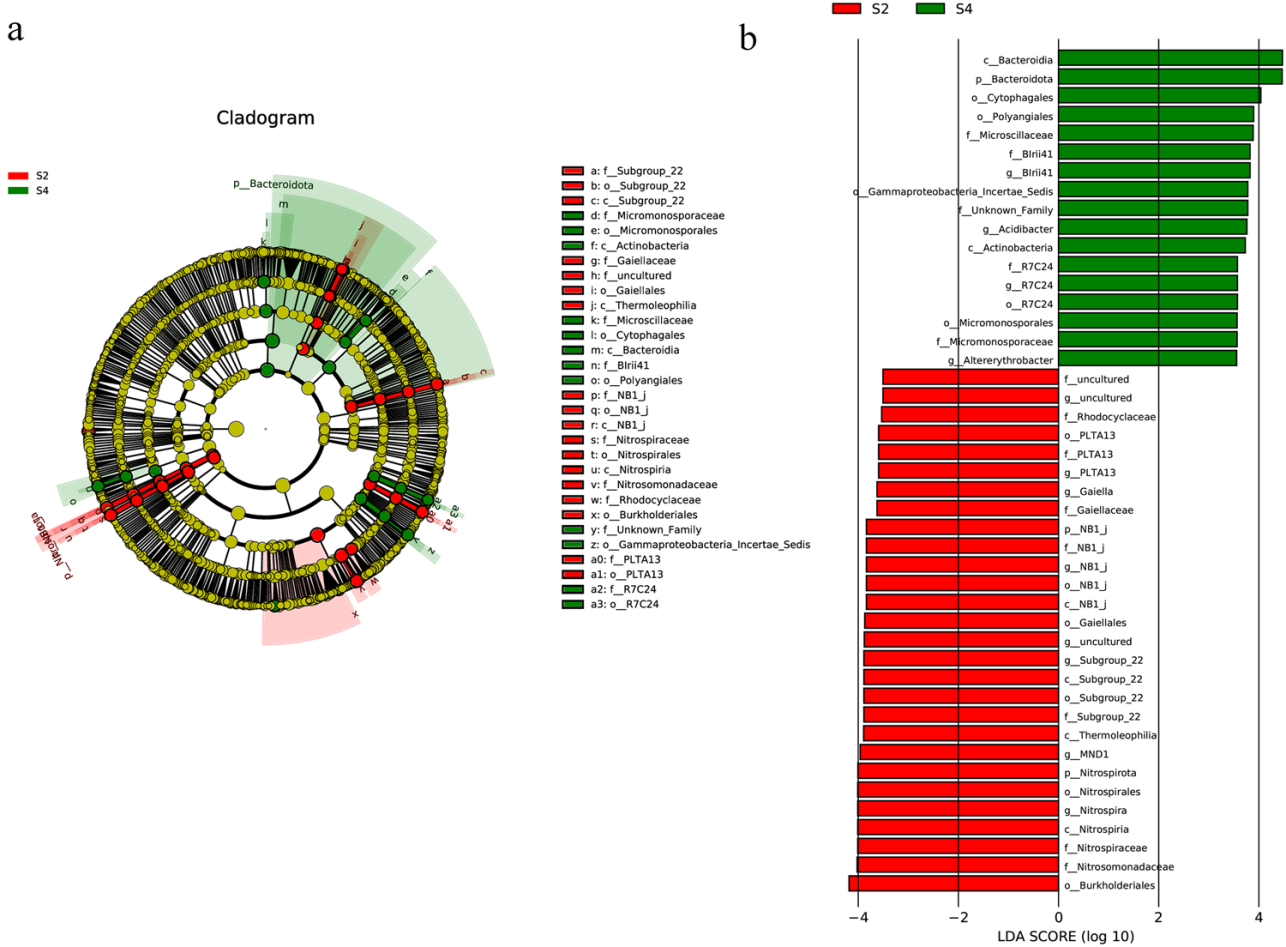
Linear discriminant analysis effect size (LEfSe) showing abundance histograms of major taxonomic units significant in S2 (Control 2) and S4 (Treat 2) rhizosphere soils. (**a**) Cladogram of bacterial communities between S2 and S4 groups. The red and green nodes indicate species with relatively high abundance in S2 and S4 samples, respectively, while the yellow nodes represent species with no significant difference between the two groups. The diameter of the nodes is proportional to relative abundance. Each layer of nodes indicates phylum/class/order/family/genus. (**b**) Linear discriminant analysis (LDA) scores presented as horizontal bars for biomarker bacteria with a score (log10) > 3.5 and an alpha value of 0.01. Green and red colors indicate S2 and S4.

To reveal the composition of different species in soil microbial communities, linear discriminant analysis Effect Size (LEfSe) analysis was employed to obtain a cladogram and calculate the linear discriminant analysis (LDA) score of differentially represented ASVs to determine the contribution size (Fig. 4a). The default LDA threshold was set at 2.0 (*P* < 0.01). The results revealed that Bacteroidetes, Bacteroidia, and Cytophagales were the most highly represented microbiota in the intercropping system. In contrast, *Burkholderiales*, *Nitrosomonadaceae*, *Nitrospira*, *Nitrospiraceae*, and *Nitrospirales* were the most abundant in the monocropping system (Fig. 4b). Together, these results suggest that changes in soil bacterial community composition are related to different planting patterns.

### Correlations among the soil physicochemical properties and bacterial communities in different cultivation patterns

**Figure 5:**
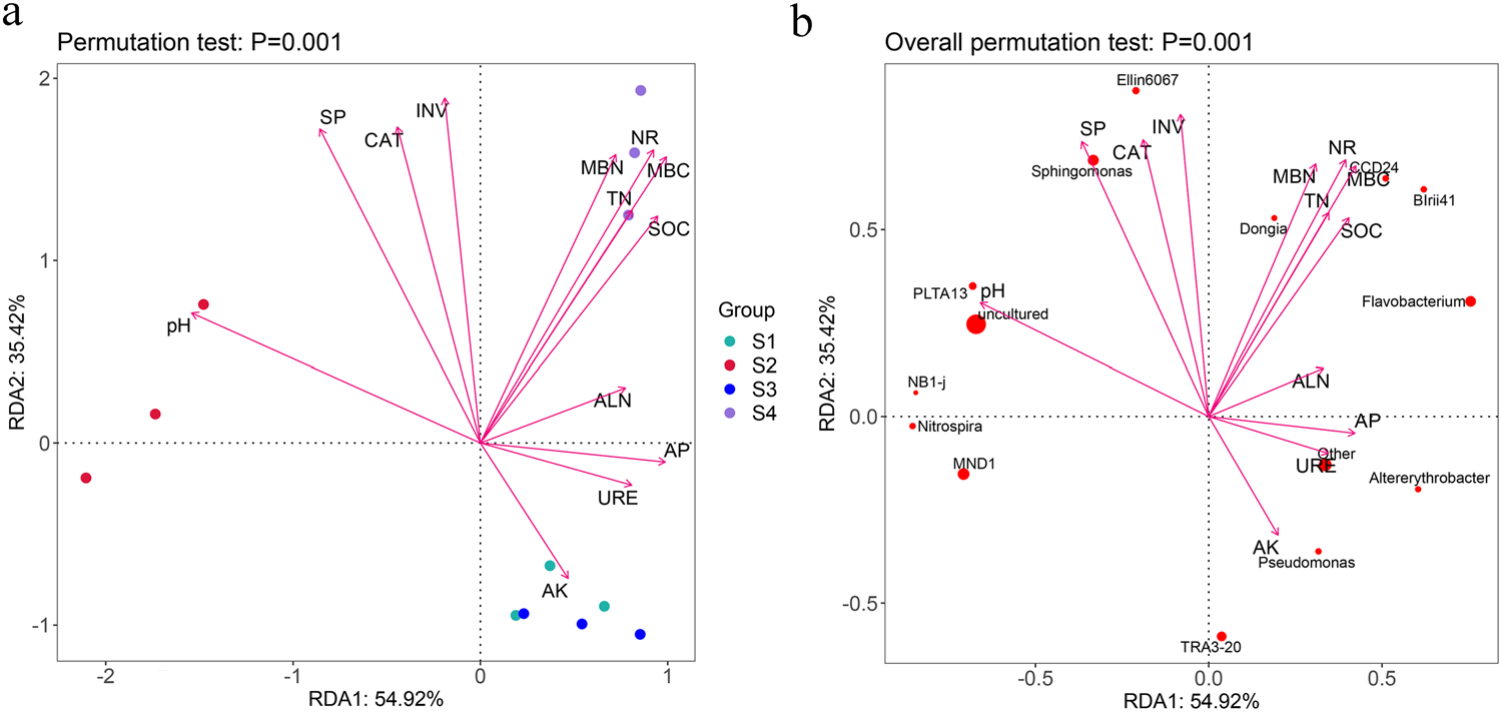
Redundancy analysis (RDA) plot. (**a**) RDA of soil properties and environmental variables; (**b**) RDA of soil bacterial community structure and environmental variables. The arrow length in the plot represents the correlation strength between environmental variables and microbes (the longer the arrow length, the stronger the correlation). The perpendicular distance between microbes and environmental variable axes reflects their correlations (the smaller the distance, the stronger the correlation). The fifteen most abundant bacterial genera are shown. S1: Control 1; S2: Control 2; S3: Treat 1; S4: Treat 2.

The redundancy analysis (RDA) described the correlation between bacterial composition, soil physicochemical indicators, and soil samples, revealing that environmental variables were responsible for 90.34% of the soil bacterial community, with 54.92% and 35.42% of the total data variation in the first and second axes, respectively (Fig. 5a,b).

The community structures of the control 1 samples were more similar to those of treat 1. The main factors influencing the intercropping system were NR, MBN, MBC, TN, and SOC, and the differences were attributed to the bacterial genera *Dongia*, *CCD24*, *Blrii41*, and *Flavobacterium*. The differences between the control 1 and 2 samples were mainly influenced by pH and were strongly correlated with *Nitrospira*, *NB1-j*, *MND1*, and *PLTA13*. In contrast, the differences between treat 1 and 2 samples were primarily influenced by AK and URE and were strongly related to *Pseudomonas*, *TRA3-20*, and *Altererythrobacter*. In addition, the environmental variables SOC, TN, MBC, MBN, NR, CAT, and pH had *p*-values < 0.01, while the *p*-values for INV and SP were < 0.001, indicating that these variables were the main factors significantly affecting the bacterial community (Table S2).

### Prediction of functional-capacity profiling of rhizosphere microbiomes in different cultivation patterns

**Figure 6:**
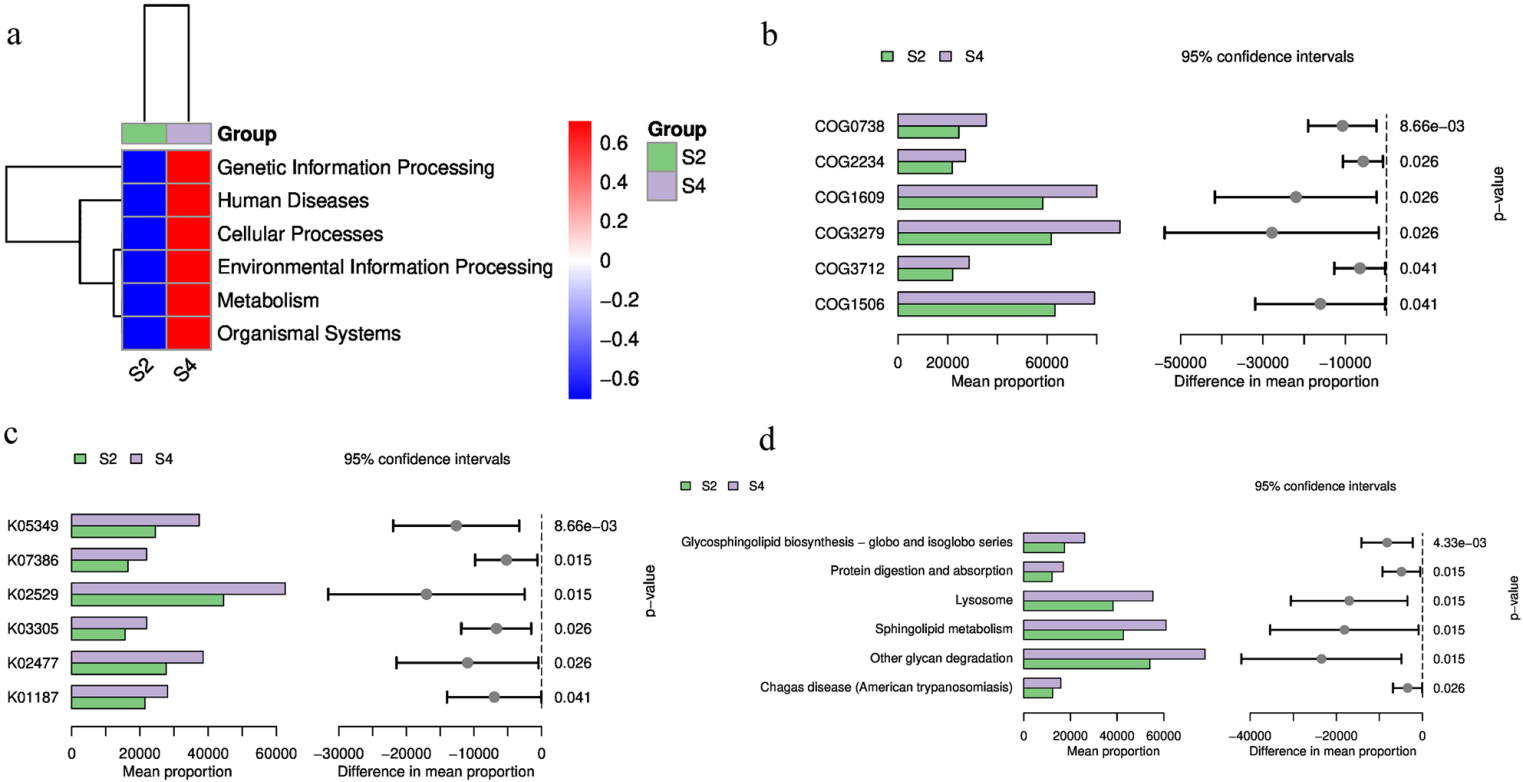
KEGG and COG-based function prediction by PICRUSt2 analysis. (**a**) Soil bacterial genes abundance at KEGG level 1. (**b**) The top 6 COG abundance proteins between Control 2 and Treat 2. (**c**) The top 6 KO abundance proteins between Control 2 and Treat 2. (**d**) The top 6 abundance of deferentially expressed bacterial proteins at KEGG level 3.

To determine the predicted metabolic functions of microbial communities, KEGG metagenome functional prediction of identified ASVs based on 16S rRNA gene sequences was performed using the Phylogenetic Investigation of Communities by Reconstruction of Unobserved States (PICRUSt) tool. A total of 51 KEGG pathways with significant differences were predicted for the intercropping planting pattern (P < 0.05). According to the PICUSt analysis, the soil microbiome of the intercropping system had a significantly different functional composition of bacterial genes compared to the control monocropping system (Fig. 6a), presenting genes related to genetic information processing, human diseases, cellular processes, environmental information processing, metabolism, and organismal systems. These results indicated that the soil microbiome was more active and diverse during kiwifruit intercropping with *A. blazei*.

Using the Wilcoxon algorithm, COG-based function prediction was attempted to obtain the top six differentially expressed proteins with significant *p*-values and highest abundance. The results indicated that fucose permease, Zn-dependent amino- or carboxypeptidase, DNA-binding transcriptional regulator, DNA-binding response regulator, periplasmic ferric-dicitrate-binding protein (FerR), and dipeptidyl aminopeptidase/acylaminoacyl peptidase were significantly enriched (Fig. 6b). Similarly, KO results showed that beta-glucosidase, endopeptidase, LacI family transcriptional regulator, proton-dependent oligopeptide transporter, two-component system, response regulator, and alpha-glucosidase were significantly enriched (Fig. 6c). Furthermore, the differentially expressed proteins were significantly enriched in glycosphingolipid biosynthesis, protein digestion and absorption, lysosomes, sphingolipid metabolism, glycan degradation, and Chagas disease pathways at KEGG level 3 (Fig. 6d).

### Metabolomics analysis

Through quality control samples and quantitative analysis of the raw data, a total of 5959 metabolites were identified in the bacterial community, of which 2286 were negative and 3673 were positive. Metabolites were subjected to multivariate analysis. PCA and OPLS-DA results indicated that the treat 2 samples were dramatically different from the control 2 samples, along with R2X = 89.3%, R2Y = 99.3%, and Q2 = 94.5% (Fig. S4 and S5a). The permutation plot of OPLS-DA showed that the intercepts of R2 and Q2 were 0.728 and -0.691, respectively (Figure S5b). All multivarite analyses showed a similar classification of the various treatments. These results suggested that intercropping had a significantly influenced the metabolic profile.

**Figure 7:**
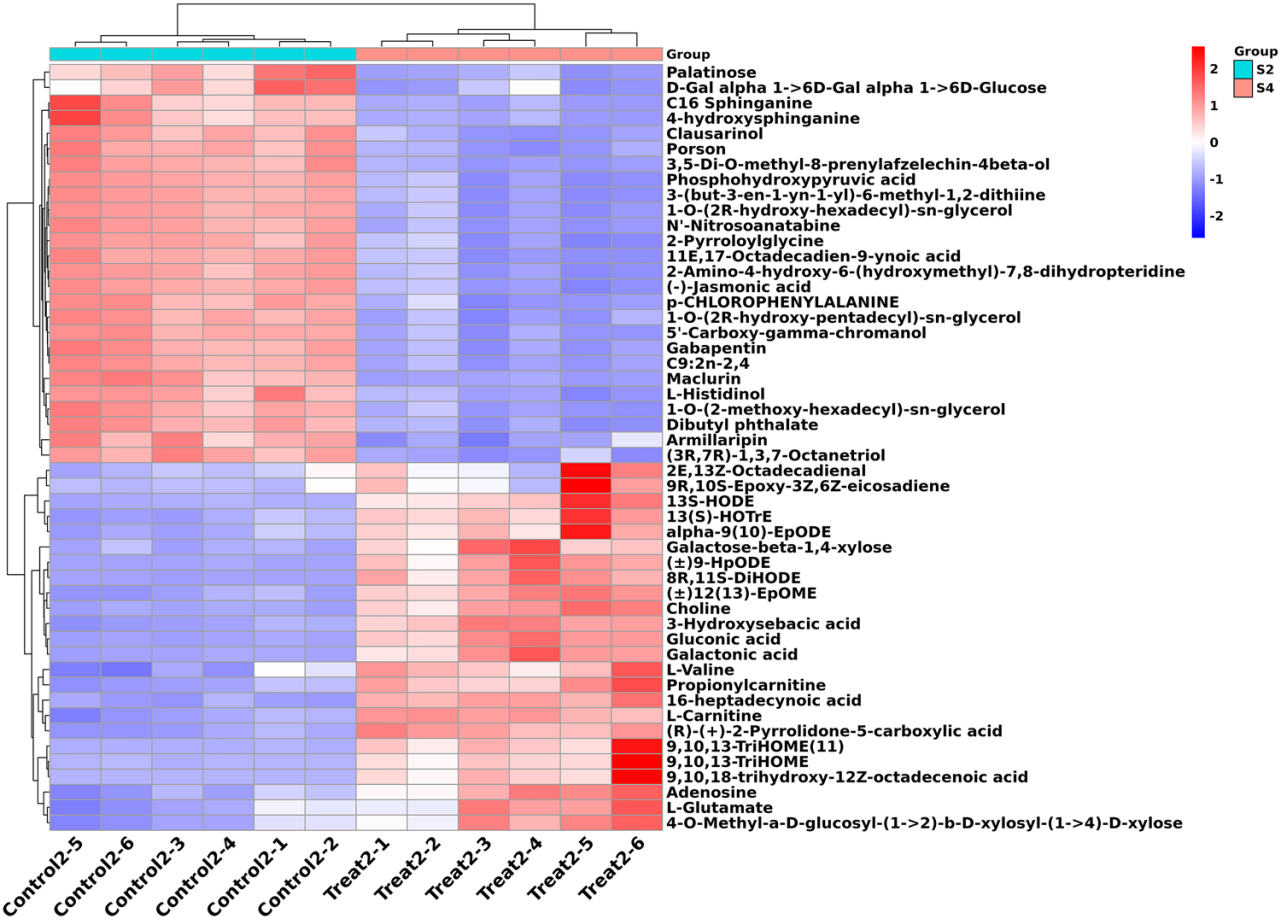
Heatmap analysis of top 50 differentially expressed metabolites between Control 2 and Treat 2. Each column indicates a sample name, and each row indicates a differentially expressed metabolite. The color gradient from blue to red represents the expression abundance of differentially expressed metabolites from low to high.

The VIP value was obtained according to the OPLS-DA model, where VIP > 1 and *p*-value < 0.05 were used as the threshold to obtain differential metabolites (DMs). A total of 358 DMs were identified between the intercropping and monocropping cultivation patterns, with an increase in 194 metabolites and decrease in 164 metabolites. Dynamic variations of these critical metabolites during the processing of the Kiwifruit/*A. blazei* intercropping system was visualized using heatmap analysis (Fig. 7). Consistent with the OPLS-DA findings, heatmap cluster analysis based on the Pearson correlation coefficient showed that the included samples could be categorized into two groups. The metabolic and functional differences between groups mainly occurred in fatty acids, glycerolipids, diarylheptanoids, organooxygen, and organonitrogen compounds. The highest number of metabolites affected by intercropping were related to lipids and lipid-like molecules. The most prominent up-regulated metabolites were zaltoprofen, quinic acid, biflorin, sporidesmolide I, and magnesium dipropionate. In addition, volcano plots of differentially expressed metabolites showed that the number of significantly up-regulated metabolites was remarkably greater than that of significantly down-regulated metabolites. These results indicated that intercropping induced a more up-regulated expression of metabolites (Figure S5).

### KEGG enrichment analysis of differential metabolic pathways

**Figure 8:**
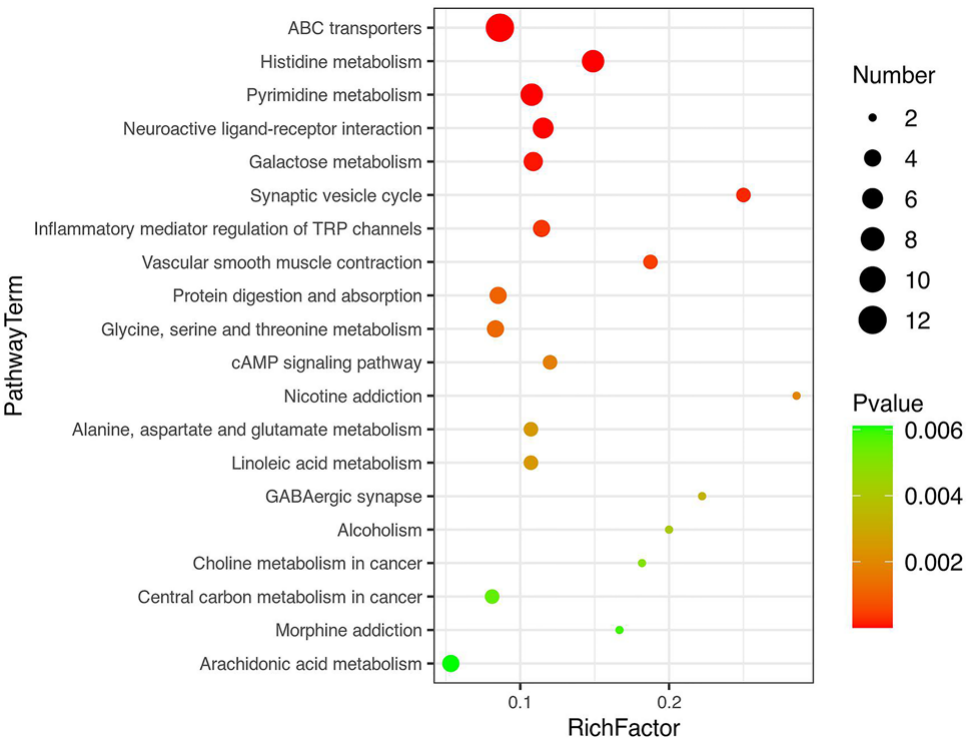
The top 20 significant pathways with up-regulated and down-regulated differential metabolites (DMs) on KEGG. The X-axis denotes the enrichment factor, and the Y-axis represents the metabolic pathway name. The bubble size reflects the number of DMs involved. The bubble color from red to green indicates the decrease in *p*-value in the same order.

A total of 63 DMs were enriched in the KEGG pathway, of which 43 were expressed up-regulated and 20 were down-regulated. Among the most enriched metabolites, fifteen metabolites belonged to fatty acyls, eleven of the metabolites consisted of carboxylic acids and derivatives, and eleven were related to organooxygen compounds (Table S3). In particular, the main enriched KEGG metabolic pathways revealed that DMs was significantly enriched in ABC transport, histidine metabolism, pyrimidine metabolism, neuroactive ligand-receptor interaction, galactose metabolism, inflammatory mediator regulation of TRP channels, protein digestion and absorption, glycine, serine, and threonine metabolism, and arachidonic acid metabolism pathways, with annotations of 12, 7, 7, 6, 5, 4, 4, 4, and 4 metabolites, respectively (Fig. 8). Additionally, kiwifruit rhizospheric soil samples showed a considerable increase in ABC transporters, which increases the possibility of the involvement of these metabolites in the transport of bioactive compounds.

### Relationships between microbial taxa and metabolites

Correlation analysis was conducted using Spearman’s algorithm for a detailed understanding of the relationship between metabolites and microbial taxa in the intercropping system. The findings showed strong correlations between the metabolic products and the dominant genera of the microbial communities in the different groups. A total of 95 bacterial genera were identified with significant correlations with 79 soil metabolites (threshold of |R|> 0.70, *P* < 0.05) (Table S4). Among the bacterial genera, *CCD24*, *Permianibacter*, *Ohtaekwangia*, *Flavobacterium*, *Altererythrobacter*, *Chryseolinea*, *Hyphomicrobium*, and *mle1-7* were the key taxa that were strongly related to soil metabolites.

**Figure 9:**
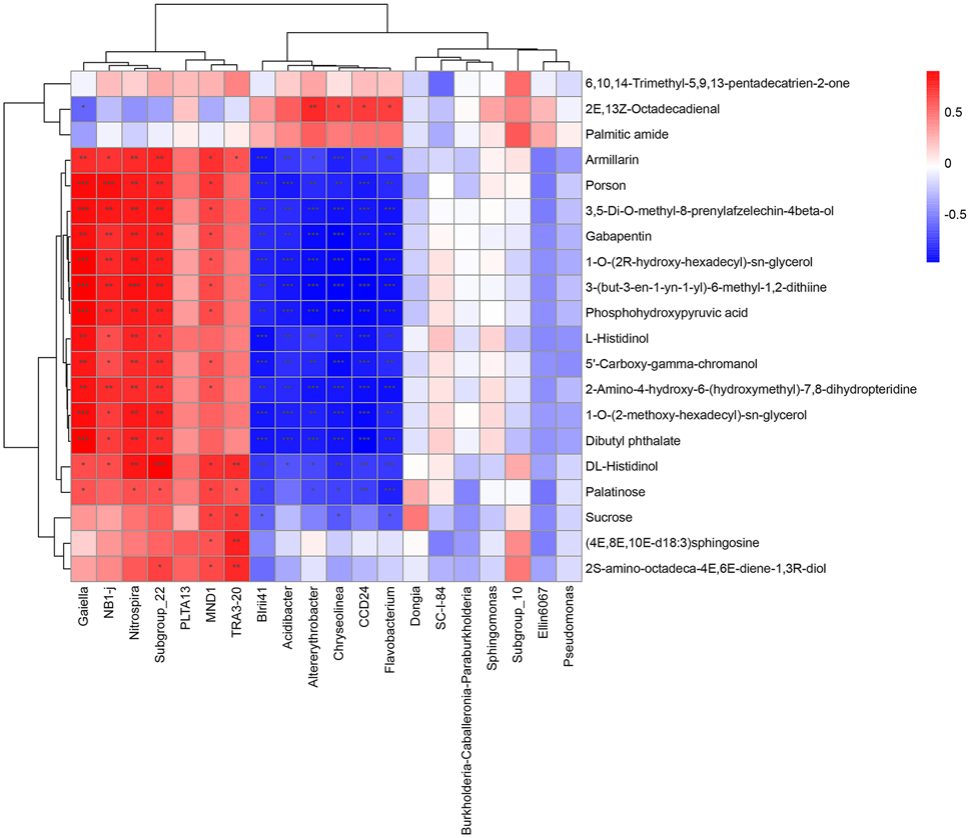
Association between metabolic products and microbial communities according to Spearman’s correlation algorithm. The horizontal axis presents the metabolic products, while the vertical axis presents the microbial communities at the genus level. Red and blue indicate positive and negative correlations, respectively. The darker the color of the squares in the graph, the more significant the correlation. ***p-value < 0.001, **p-value < 0.01, and *p-value < 0.05.

As depicted in Fig. 9, the bacterial genera *Gaiella*, *NB1-i*, *Nitrospira*, and *Subgroup_22* were positively correlated with the metabolites armillarin, porson, gabapentin, phosphohydroxypyruvic acid, L-histidinol, 5′-carboxy-gamma-chromanol, dibutyl phthalate, and DL-histidinol. Similarly, significant positive correlations were observed between these metabolites and the bacterial genera *BIrii41*, *Acidibacter*, *Altererythrobacter*, *Chryseolinea*, *CCD24*, and *Flavobacterium*. Therefore, metabolic activities in the soil were closely related to the microbial communities.

## Discussion

Multi-species cropping is an ecological planting strategy essential for food production and sustenance worldwide. In addition to controlling the microbial community in the rhizosphere, it also plays a significant role in enhancing the soil quality. The variety and organization of the rhizosphere microbiome can have a significant impact on plant health and soil-borne illnesses. In this study, 16S rRNA gene sequencing and LC–MS untargeted metabolic profiling analyses were conducted for monocropping and intercropping. The results indicated that physicochemical characteristics and microbial diversity were improved in Kiwifruit/*A. blazei* intercropping system compared to the monocropping system.

### Intercropping improves kiwifruit productivity

To date, most plant microbe studies have focused on identifying and manipulating specific plant-beneficial microbes such as plant growth-promoting rhizobacteria (PGPR)^33^. Despite the importance of rhizosphere microbes, the combined effect and relative importance of the diverse factors that affect rhizosphere microbial community structure remain undetermined^34^. With the recent development of next-generation sequencing and other omics technologies, new insights into soil microbial ecology have been provided, particularly in the identification of microbial associations and traits^35^. However, the molecular mechanisms specific to PGRP factors controlling different aspects of rhizosphere microbes in kiwifruit remain poorly understood.

Previous studies have shown that using soil surface cover crops as an intercrop in orchards can substantially improve nitrogen and organic carbon content, water utilization, and yield^36–38^. Additionally, cover crop intercropping can also provide defense against pest and disease invasions^39^. These limiting factors are essential for enhancing the orchard yields. In this study, we demonstrated that intercropping with *A. blazei* significantly increased kiwifruit yield and improved various physiological and biochemical indices. For example, kiwifruit quality improved with a considerable increase in vitamin C content, soluble solids, dry matter, and sugar-to-acid ratio (*p* < 0.05) (Table 4). Furthermore, the ability of the root system to absorb various elements of the soil was markedly increased, facilitating the full use of soil elements. Hence, *A. blazei* mushrooms provide several elements necessary for kiwifruit growth. Additionally, the yield of the edible mushroom *A. blazei* was enhanced; however, detailed physiological data were not determined. In addition, the Shannon index was positively linked to the diversity of the soil bacterial community, revealing the evenness of the soil bacterial community. Meanwhile, diversity indices, including Chao1 and ACE, indicate the richness of the soil bacterial community^40^. A significant increase in diversity indices in our study further suggested that intercropping of *A. blazei* with kiwifruit had a long-lasting beneficial influence on the ecological environment and microbial diversity of the soil.

**Table 4:** Determination of kiwifruit quality in crop monoculture (CK) and intercropping with *Agaricus blazei* (IA).

Samples	Single fruit weight	Glycolic acid ratio	Dry matter	Vitamin C	Soluble solids
	g	%	%	mg 100 g^−1^	%
CK	86.09 ± 5.79 b	18.57 ± 2.30 b	16.82 ± 0.63 b	95.91 ± 1.94 b	12.54 ± 0.21 b
IA	94.85 ± 9.05 a	24.93 ± 2.94 a	18.73 ± 0.84 a	109.96 ± 4.77 a	14.86 ± 0.63 a

*CK* control samples, *IA* treated samples. Different letters present significant differences (*p* < 0.05). Values represent the mean ± standard deviation of three replicates.

### Intercropping influences soil microbial community structure

Intercropping can promote plant development by stimulating the growth of many beneficial soil bacterial phyla, such as Proteobacteria, Bacteroidetes, Gemmatimonadota, Acidobacteriota, Myxococcota, and Actinobacteria^41–43^. For example, Proteobacteria utilize a variety of carbon sources for rapid growth, which is positively correlated with soil carbon content^44^, wheres Myxococcota was positively correlated with total soil nitrogen^45^. Moreover, Bacteroidetes often acts as a crucial indicator of soil fertility and quality, ultimately influencing crop productivity^46^. Consistent with previous reports, the predominant bacterial phyla in our study included Proteobacteria, Bacteroidetes, Myxococcota, and Patescibacteria, exhibiting a robust distribution and dramatic increase in soil TN and SOC content. In addition, the total number of ASVs was markedly elevated, which corroborates the results of the significantly higher levels of physiological indicators of total microorganisms, MBC, and MBN. Furthermore, intercropping enhanced the activity of soil enzymes such as URE and NR, which are involved in soil nutrient cycling and metabolic capacity in kiwifruit orchards. Taken together, our findings provide evidence that kiwifruit/*A. blazei* intercropping can alter soil microbial community structure and improve fertility. An increase in soil nutrient contents can significantly enhance plant growth and disease resistance. Our findings imply that different plant communities can recruit of plant beneficial-bacteria as a mechanism for disease suppression.

### Intercropping increases the number of beneficial *bacteria*

Planting cover crops in orchards can increase soil surface cover, providing favorable environmental conditions for promoting the growth and reproduction of microorganisms and increasing the amount and diversity of soil microbes^47,48^. The rhizospheric microorganism *Acidibacter* plays a vital role in promoting plant growth and controlling plant diseases^49^ and is an important indicator of soil nutrient properties^50^. An increasing number of macrofungi have shown heavy metal enrichment capacity, especially the mushroom genus *Agaricus*^51,52^. In addition, bacterial genera such as *BIrii41* and *Altererythrobacter* exhibit heavy metal tolerance^53^. In this study, the most abundant bacterial genera in the intercropping system were *Flavobacterium*, *BIrii41*, and *Acidibacter*, with higher relative abundances of the beneficial genera *BIrii41*, *Acidibacter*, and *Altererythrobacter* than in the monocropping system. Therefore, *A. blazei* intercropping may mitigate the potential toxicity of heavy metals to kiwifruits and could potentially be applied in areas contaminated with heavy metals.

LEfSe analysis showed that Bacteroidota, Bacteroidia, and Cytophagales were significantly higher in the rhizosphere soil of the intercropping system (*p* < 0.01). Cytophagales are mainly enriched in the rhizosphere and produce enzymes that increase total organic carbon and nitrogen content, thus benefiting plant growth^54^. Conversely, the monocropping system showed a prominent abundance of Nitrospora and genera belonging to Nitrosomonadaceae and Nitrospiraceae in rhizosphere soil.

### Intercropping influences ABC transport

A wide range of compounds comprise the metabolic profiles of root-associated soils, making the root zone a crucial microhabitat that attracts specific microbial species to establish intricate connections with plants^55^. However, insufficient information is available on how intercropping affects soil metabolites produced by cover crops in kiwifruit orchards. Here, the metabolites from fatty acids, glycerolipids, diarylheptanoids, organonitrogen, and organooxygen compounds showed notable changes, which is in line with the results of soil physicochemical properties in the intercropping system. Previous studies have shown that sugarcane/peanut intercropping increases the lipid and phenolic acid contents of soils^56^. In agreement with these studies, fatty acids, carboxylic acids and derivatives, and organooxygen compounds were significantly enhanced in the intercropping system. In addition, the TN and SOC contents in the rhizosphere soil also increased, mediated by the regulated production of organonitrogen and organooxygen compounds.

ABC transport proteins are involved in transporting a wide variety of molecules across the cell membranes^57^. In soil microorganisms, ABC transporters can execute several functions, including uptake of nutrients from the soil, excretion of waste products, and transport of signaling molecules^58,59^. Moreover, ABC transporters are involved in the detoxification of toxins and other harmful substances that are possibly present in the soil by actively transporting these substances out of the cell and protecting microorganisms^60,61^. In the present study, the metabolic pathways involved in ABC transport were significantly enriched in the intercropping system. Additionally, KEGG enrichment analysis revealed that intercropping enhanced bacterial metabolism, which is related to the resistance of kiwifruit root systems to soil-borne illnesses. Thus, ABC transporters are essential for bacterial growth and survival and may confer an advantage to bacterial communities in resisting pathogens.

Thus, our study provides a mechanistic explanation of kiwifruit-*A. blazei* intercropping-mediated efficient generation of rhizosphere-beneficial bacteria to improve fruit yield and prevent soil-borne diseases. Furthermore, this study demonstrated that crop intercropping can control microbial populations to benefit plant health.

## Conclusions

In this study, we used 16S rRNA and soil microbial metabolome sequencing to investigate the soil bacterial community structure and diversity in an intercropping system comprising *A. blazei* and kiwifruit. The results showed that cover crops significantly increased various soil parameters, including ALN, AP, AK, TN, SOC, MBC, and MBN, and altered the bacterial abundance and community composition. Interestingly, we observed a shift in the bacterial taxa with mostly plant-beneficial properties. Based on these findings, we conclude that cover crops can influence the soil environment in a differential manner and regulate the soil microbial community composition to benefit crop production. In addition, we identified several DMs involved in the synthesis of fatty acids, carboxylic acids, and organooxygen compounds. The DMs were significantly enriched in ABC transport, histidine metabolism, and pyrimidine metabolism. Overall, our results suggested that kiwifruit-*A. blazei* intercropping can serve as an effective and sustainable strategy for maximizing the economic and environmental benefits of kiwifruit plantations.

### Supplementary Information

Supplementary Figure S1.

Supplementary Figure S2.

Supplementary Figure S3.

Supplementary Figure S4.

Supplementary Figure S5.

Supplementary Figure S6.

Supplementary Legends.

Supplementary Table S1.

Supplementary Table S2.

Supplementary Table S3.

Supplementary Table S4.

## Data Availability

The original contributions presented in the study are included in the article/Supplementary Material. Further inquiries can be directed to the corresponding author.
