# Author Correction: Kiwifruit-*Agaricus blazei* intercropping effectively improved yield productivity, nutrient uptake, and rhizospheric bacterial community

**DOI:** 10.1038/s41598-024-69709-5

**Published:** 2024-08-14

**Authors:** Chuan Shen, Xia Li, Jianfeng Qin

**Affiliations:** 1 Shaannan Eco-Economy Research Center, Ankang University, Ankang, 725000 China; 2 Department of Electronic and Information Engineering, Ankang University, Ankang, 725000 China; 3 Ankang Academy of Agricultural Sciences, Ankang, 725000 China

Correction to: *Scientific Reports* 10.1038/s41598-024-66030-z, published online 17 July 2024

In the original version of this Article, Affiliation 1 was incorrect.

“Shaannan Eco-Economy Research Center, Ankang College, Ankang, 725000, China.”

now reads

“Shaannan Eco-Economy Research Center, Ankang University, Ankang, 725000, China.”

The original Article has been corrected.

