# Supplementary figures and images for: Kiwifruit-Agaricus blazei intercropping effectively improved yield productivity, nutrient uptake, and rhizospheric bacterial community
Source: Sci Rep. 2024 Jul 17;14:16546. doi: 10.1038/s41598-024-66030-z (PMC11255323; doi:10.1038/s41598-024-66030-z)

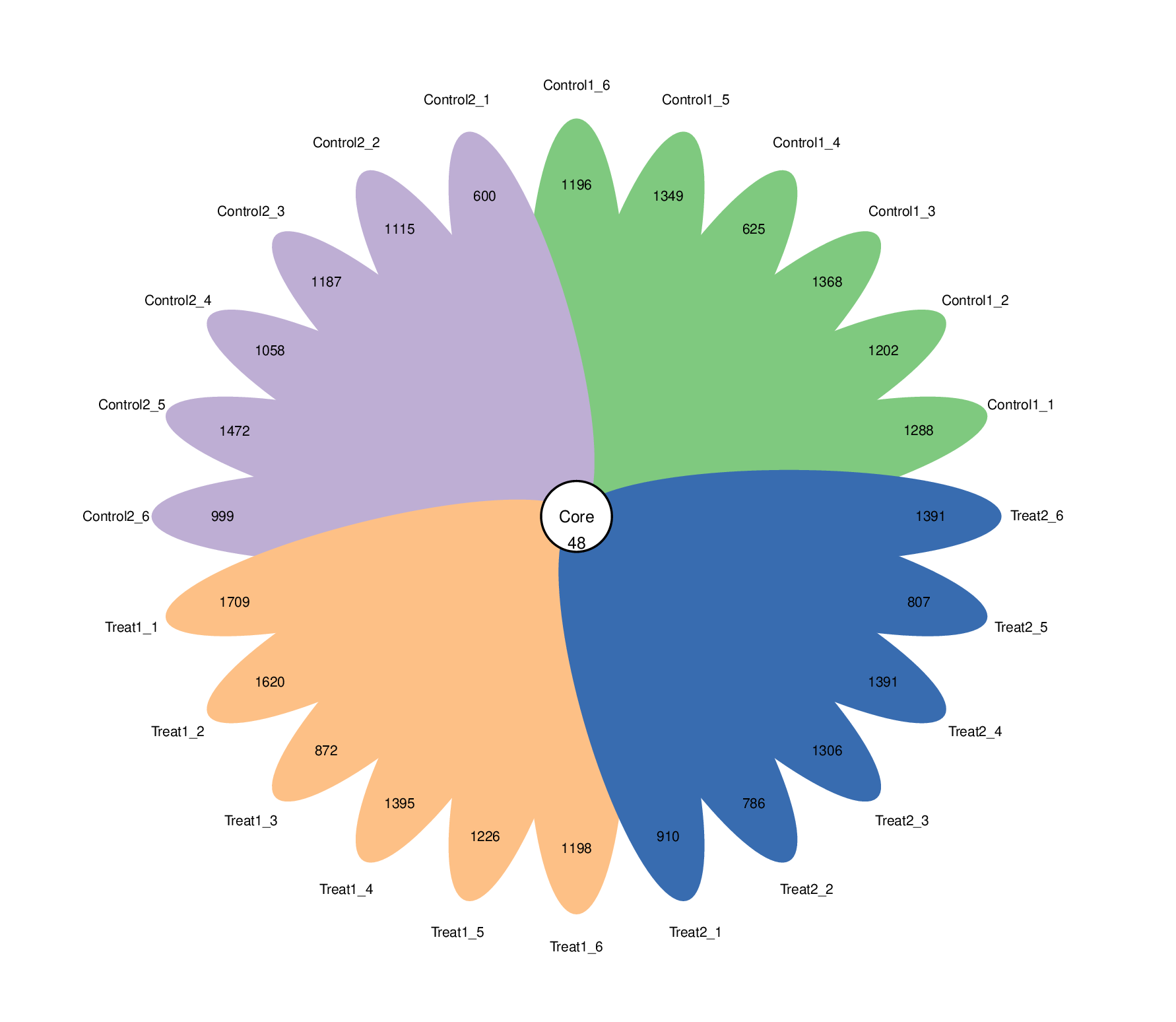

Supplement: Supplementary file 1 — Supplementary Figure S1. [file 41598_2024_66030_MOESM1_ESM.tif]

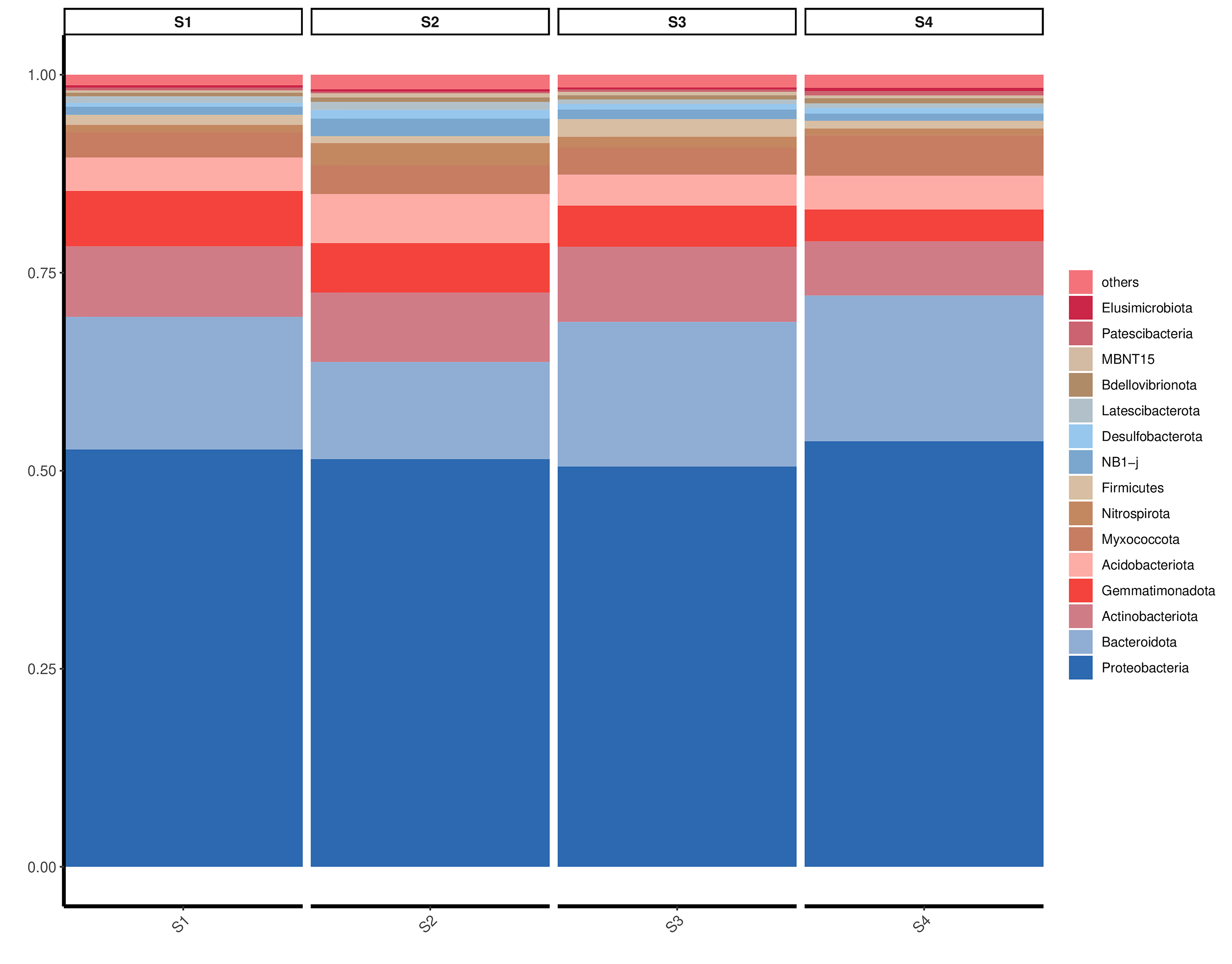

Supplement: Supplementary file 2 — Supplementary Figure S2. [file 41598_2024_66030_MOESM2_ESM.tif]

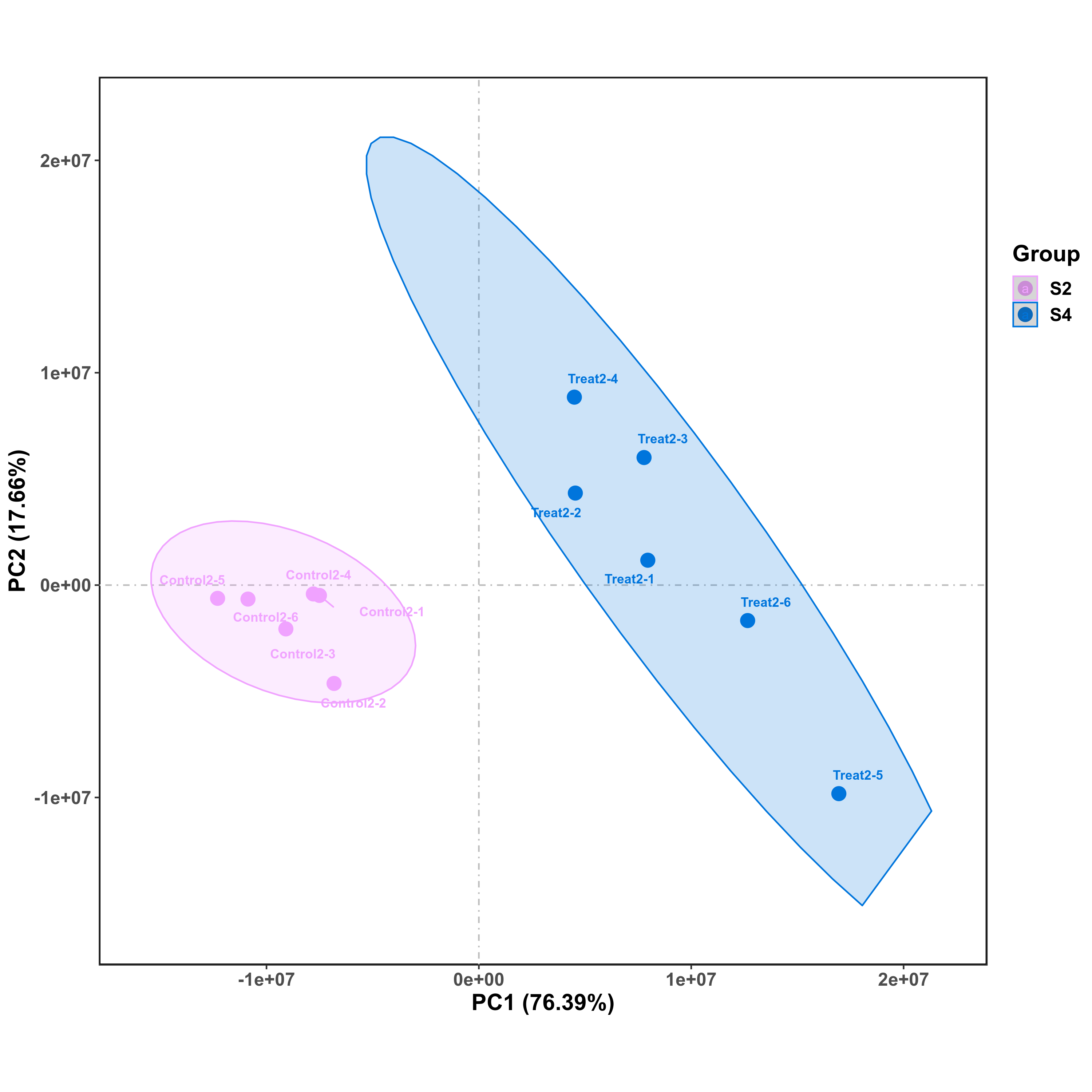

Supplement: Supplementary file 4 — Supplementary Figure S4. [file 41598_2024_66030_MOESM4_ESM.tif]

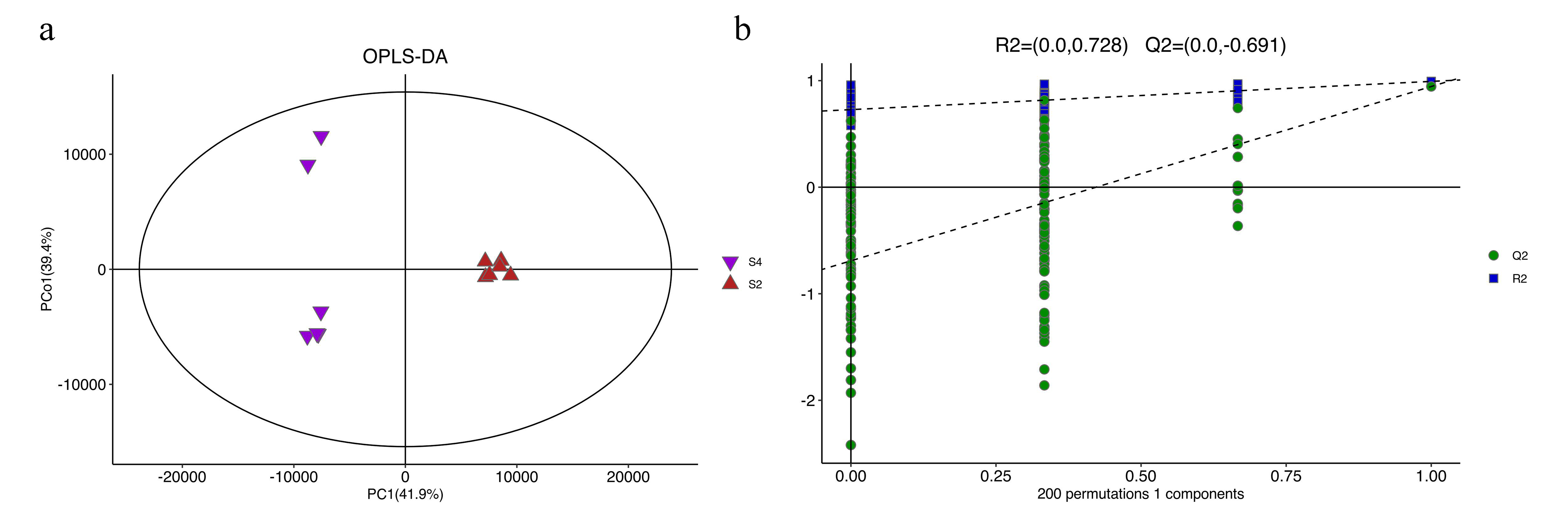

Supplement: Supplementary file 5 — Supplementary Figure S5. [file 41598_2024_66030_MOESM5_ESM.tif]

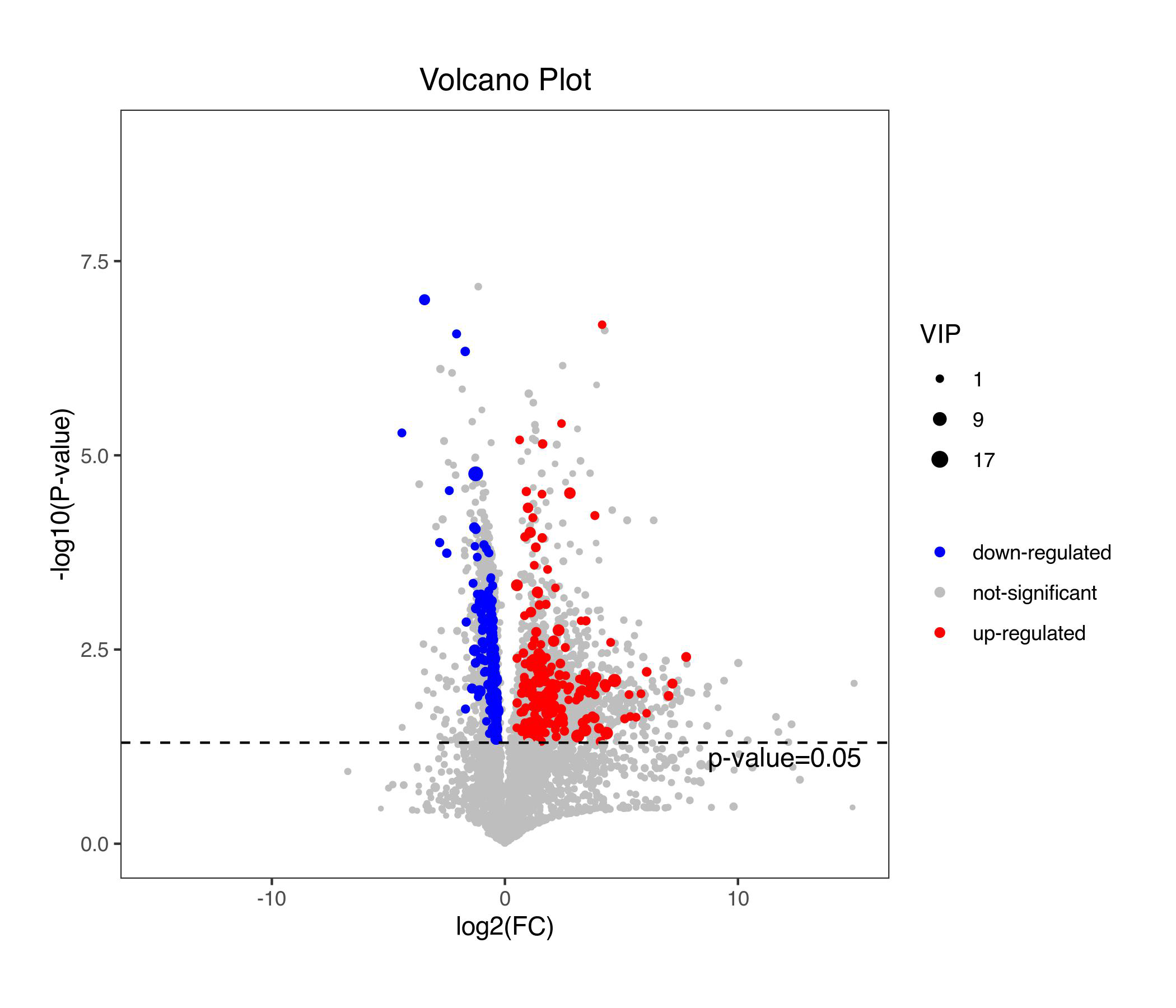

Supplement: Supplementary file 6 — Supplementary Figure S6. [file 41598_2024_66030_MOESM6_ESM.tif]
