# Supplementary material for: Kiwifruit-Agaricus blazei intercropping effectively improved yield productivity, nutrient uptake, and rhizospheric bacterial community
Source: Sci Rep. 2024 Jul 17;14:16546. doi: 10.1038/s41598-024-66030-z (PMC11255323; doi:10.1038/s41598-024-66030-z)
Supplement: Supplementary file 7 — Supplementary Legends. [file 41598_2024_66030_MOESM7_ESM.docx]

Fig S1. Petal diagram of the distribution of ASVs. The numbers in the Core represent the ASVs common to all samples (Core ASVs), and the numbers on the petals represent the total ASVs of each sample minus the number of common ASVs.

Fig S2. Taxonomic classification of bacterial reads amplified from the rhizospheric soils of monocropping and intercropping cultivation patterns. S1: Control 1; S2: Control 2; S3: Treat 1; S4: Treat 2.

Fig S3. The top10 significant differences genus between group2 and group4.

Fig S4. The PCA analysis showed the samples between group2 and group4.

Fig S5. Multivariate analysis of Control 2 and Treat 2 samples. a: Orthogonal partial least-squares-discriminant analysis (OPLS-DA) score plot. b: Permutation plot of OPLS-DA.

Fig S6. The volcano showing the expression of differential metabolites between group2 and group4.

Table S1. Overview of 16S microbiome sequencing data from 24 samples.

Table S2. Results of RDA analysis of soil physical and chemical properties.

Table S3. All differentially expressed products obtained by metabolome sequencing.

Table S4. Results of correlation analysis of metabolites and microorganisms.
